# Single-dose prophylactic ibuprofen therapy for patent ductus arteriosus in preterm infants

**DOI:** 10.1097/MD.0000000000029915

**Published:** 2022-08-05

**Authors:** Chae Young Kim, Sung-Hoon Chung

**Affiliations:** Department of Pediatrics, Kyung Hee University School of Medicine, Seoul, Korea.

**Keywords:** ibuprofen, intraventricular hemorrhage, morbidity, patent ductus arteriosus, premature infant

## Abstract

This study aimed to evaluate the short-term morbidities and efficacy of single-dose prophylactic intravenous ibuprofen for patent ductus arteriosus (PDA) on the first day of life in preterm infants.

Data of 69 preterm infants with birth weight < 1250 g and gestational age < 30 weeks admitted to the neonatal intensive care unit were analyzed. Of these, 37 infants were assigned to the prophylactic treatment (PT) group and 32 were assigned to the nonprophylactic treatment (non-PT) group. Only the PT group administered intravenous ibuprofen (10 mg/kg) once within 6 hours after birth.

Until postnatal day 7, ductal closure occurred in 11 (34.4%) infants in the non-PT group, and in 35 (94.6%) infants in the PT group, of which 30 (81.1%) infants had ductal closure on postnatal day 1. There were 2 (5.4%) infants in the PT group and 9 (28.1%) in the non-PT group who needed ibuprofen treatment due to moderate-to-large PDA after postnatal day 7. Preterm infants in the PT group were less likely to develop an intraventricular hemorrhage (≥grade 2) (adjusted odds ratio 0.007, 95% confidence interval 0.01–0.45), had a shorter duration of invasive ventilatory support and central venous catheter, and earlier postnatal age to achieve feeding of 50 and 100 mL/kg/day compared with those in the non-PT group.

Single-dose prophylactic intravenous ibuprofen on the first day of life decreased the occurrence of a persistent PDA and intraventricular hemorrhage (≥grade 2), and reduced the duration of invasive ventilatory support, central venous catheter use, and hospital stay.

## 1. Introduction

The ductus arteriosus is a blood vessel that plays an important role in fetal circulation,^[1]^ and ductal closure occurs after birth due to changes in systemic oxygen tension and prostaglandin E2.^[2]^ In term infants, the ductus arteriosus normally constricts and functionally closes by 72 hours after birth, but ductal closure is delayed in preterm infants and the risk of a patent ductus arteriosus (PDA) is inversely proportional to gestational age (GA).^[3]^ While the use of pharmacologic intervention in very low BW infants (VLBWIs, BW < 1500 g) with PDA has decreased recently,^[4,5]^ more substantial studies are required before noninterventional approaches can be widely adopted, because a hemodynamically significant PDA has been associated with intraventricular hemorrhage (IVH), bronchopulmonary dysplasia (BPD), and necrotizing enterocolitis (NEC).^[6–8]^

Various therapeutic options have been discussed, including prophylactic treatment (PT) with cyclooxygenase (COX) inhibitors.^[9]^ Although prophylactic administration of COX inhibitors such as indomethacin and ibuprofen reduces the incidence of severe IVH (grades 3–4), it has no effect on mortality, BPD, NEC, and time to reach full feeds. Prophylactic COX inhibitors are no longer recommended because they have several adverse effects including oliguria, gastrointestinal (GI) bleeding and perforation, platelet dysfunction, and thrombocytopenia.^[10–13]^ Major issues remain to be clarified, both with respect to diagnosis and treatment and the detailed risks and benefits of available treatment alternatives are still under investigation. All previous randomized controlled trials that examined the effects of prophylactic ibuprofen treatment used 3 doses of ibuprofen for 3 days after birth.^[14–19]^

Our practice was to perform conservative management of the PDA until 7 days after birth, and ibuprofen therapy was initiated if the PDA remained hemodynamically significant and persisted beyond 7 days of life. However, after July 2017, all VLBWIs in our neonatal intensive care unit (NICU) were administered a single dose of prophylactic ibuprofen (10 mg/kg) within 6 hours of birth. This study aimed to evaluate the efficacy of single-dose prophylactic intravenous (IV) ibuprofen on the first day of life in very preterm infants.

## 2. Methods

### 2.1. Study design

**Figure 1.**
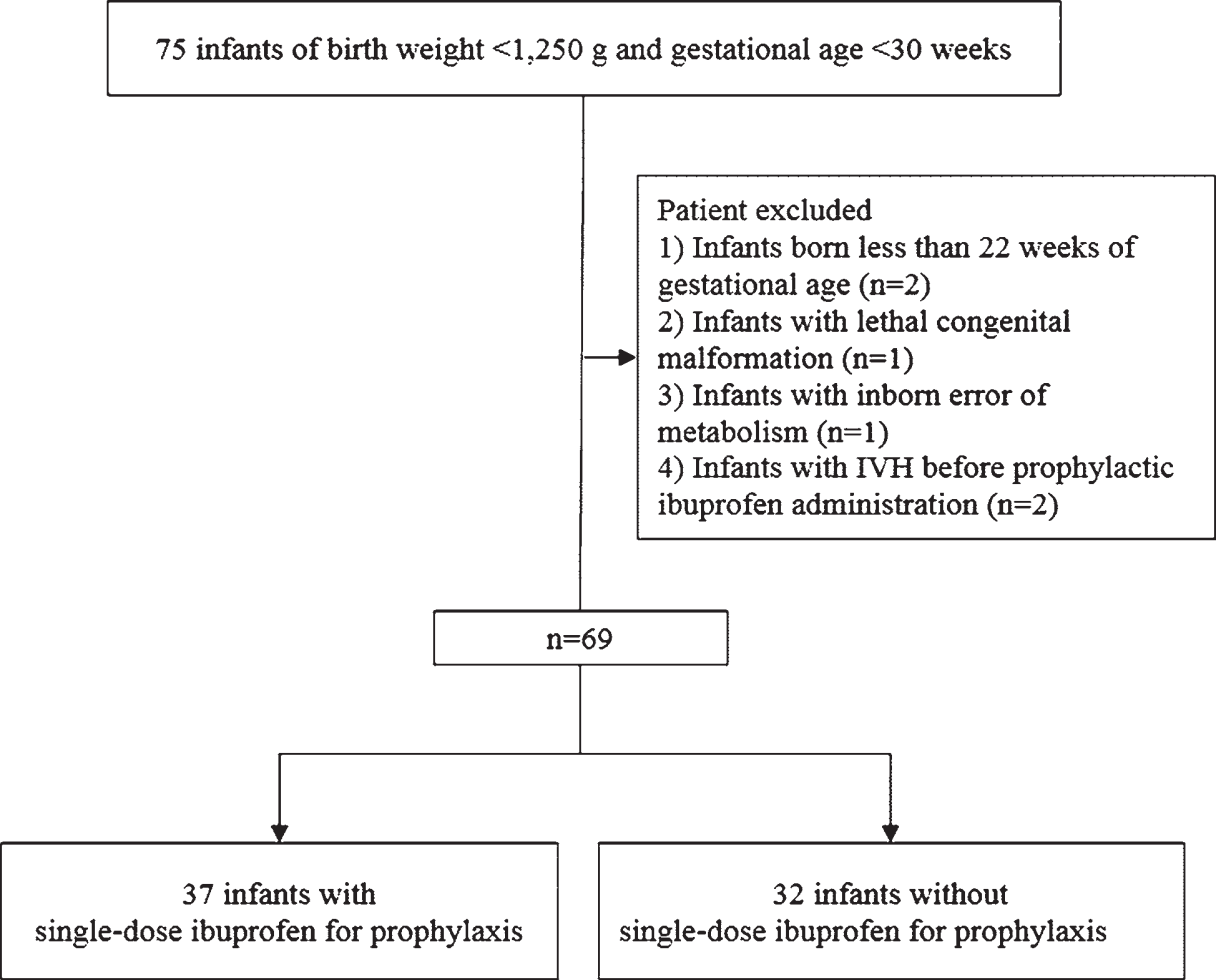
Flow chart of participants.

In this retrospective study, we reviewed 75 infants with birth weight (BW) < 1250 g and GA < 30 weeks, who were born alive and admitted to the NICU of the Kyung-Hee University Hospital at Gangdong, Korea, between January 2016 and July 2019. The institutional review board waived the requirement for informed consent for this retrospective chart review (approval number: KHNMC 2020-07-009). The exclusion criteria were birth at <22 completed weeks of gestation, lethal congenital malformations, inborn errors of metabolism, and IVH before prophylactic ibuprofen administration (Fig. 1). We maintained similar policies regarding ventilatory support and fluid management, nutrition, hemodynamic management, and indications for hospital discharge except for PDA management, from 2016 to 2019.

### 2.2. Ibuprofen administration and echocardiography

From January 2016 to June 2017, none of the preterm infants received PDA treatment for up to 1 week after birth, regardless of the PDA size. Echocardiography was performed after the first week to determine whether treatment was necessary. However, following this protocol, extremely low BW infants (BW < 1000 g), especially born at 22 to 25 weeks of gestation, showed a tendency for increased pulmonary hemorrhage within 1 week of life, which may be due to PDA. Therefore, from July 2017, all VLBWIs were administered a single dose (10 mg/kg) of prophylactic ibuprofen lysine (Arfen, Lisapharma, Erba, Italy) within 6 hours of birth if there were no contraindications. After administration, an echocardiogram was performed every 24 hours until the PDA closed or became (meaning “almost impossible to measure”) and repeated at the end of the 1st week. Infants with a moderate-to-large sized, hemodynamically significant PDA after the first week received IV ibuprofen using a syringe pump over 15 minutes, at a dose of 10 mg/kg, followed by 5 mg/kg after 24 and 48 hours, if there were no contraindications for pharmacologic treatment such as NEC, decreased urine output, or bleeding tendency. This course was followed by a second and third cycle in the nonresponder infants. PDA ligation was performed when the ibuprofen treatment failed. One neonatologist performed echocardiography, and a moderate-to-large sized, hemodynamically significant PDA was defined as a PDA with an internal diameter that exceeds 1.4 mm and a PDA: left pulmonary artery diameter ratio of ≥0.5. Additional criteria included one or more of the following: (1) reversed diastolic flow in the descending aorta, (2) ductus flow velocity ≤2.5 m/s, and (3) left atrium-to-aortic root ratio ≥1.6.^[20–22]^

### 2.3. Data collection

Maternal data included maternal age, presence of oligohydramnios or polyhydramnios, gestational or overt diabetes mellitus, pregnancy-induced or pre-existing maternal hypertension (HTN), chorioamnionitis, preterm premature rupture of membranes, antenatal steroid use, delivery mode, and multiple gestations. Neonatal variables included sex, GA, BW, size in relation to GA, and Apgar scores. Neonatal morbidities before discharge and the hospital course included the occurrence of pneumothorax, respiratory distress syndrome, duration of invasive ventilatory support and central venous catheter use, postnatal age at achieving enteral feeding of 100 mL/kg, treatment for patent PDA, late-onset sepsis (LOS), NEC (≥stage 2), BPD at 36 weeks of postmenstrual age (PMA), retinopathy of prematurity (≥stage 2), IVH (≥grade 2), average length of hospital stay, and mortality. Moderate BPD was defined as the need for oxygen for >28 days plus <30% oxygen at 36 weeks of PMA according to the National Institute of Child Health and Human Development Workshop severity-based diagnostic criteria.^[23]^ NEC was defined according to the modified Bell staging classification grade of ≥II.^[24]^ Body weight, head circumferences, and lengths measured at birth and discharge were compared. In addition, we used the 2013 Fenton growth charts to determine the proportion of infants who were small for GA in the study population. All relevant data are within the paper and its Supporting Information files.

### 2.4. Statistical analysis

All analyses were performed using SPSS software (version 25.0; IBM Corp., Armonk, NY, USA). Continuous variables of both patient groups were compared using Student t-test or Mann–Whitney *U* test, and the results were expressed as mean ± standard deviation. Categorical variables were compared using the chi-square test or Fisher exact test, and the results were expressed as numbers and percentages. Stepwise multivariate analyses were used to evaluate the relationship between different outcome measures and the effect of single-dose prophylactic ibuprofen after adjustment for chorioamnionitis, multiple gestations, and a 5-min Apgar score ≤ 3. Statistical significance was set at *P* value < .05. All relevant data are within the paper and its Supporting Information files.

## 3. Results

After application of the inclusion and exclusion criteria, 69 infants were analyzed, of which 67 were discharged alive, and 2 died in the NICU. They were classified into 2 groups: the PT group consisted of 37 infants and the nonprophylactic treatment (non-PT) group consisted of 32 infants.

### 3.1. Perinatal characteristics of the PT and non-PT groups

A comparison of the perinatal characteristics between the PT and non-PT groups is shown in Table 1. There were no significant differences in perinatal characteristics (in vitro fertilization, abnormal amniotic fluid, maternal diabetes mellitus/hypertension, chorioamnionitis, preterm premature rupture of membranes, use of antenatal steroids, mode of delivery, multiple gestations, sex, small for GA, mean GA at birth, mean BW, 5-minute Apgar score ≤ 3, and use of surfactant) between the 2 groups that could have influenced the morbidity, mortality, or clinical course of preterm infants. The mothers who belonged to the PT group were older than those in the non-PT group (34.2 ± 4.4 vs 31.6 ± 3.3 years; *P* = .008). In this study, 1 infant in each group had a 5-minute Apgar score ≤ 3, but both infants recovered to a 10-minute Apgar score of 7 or higher, and a head ultrasound performed immediately after birth showed no IVH, so they were included in this study.

**Table 1:** Comparison of perinatal characteristics in the PT and non-PT groups.

Characteristics	PT group (n = 37)	non-PT group (n = 32)	*P*
Maternal age, yrs	34.2 ± 4.4	31.6 ± 3.3	.008
In vitro fertilization	7 (18.9)	6 (18.8)	.986
Oligohydramnios/polyhydramnios	5 (13.5)	5 (15.6)	.804
Diabetes mellitus	4 (10.8)	3 (9.4)	1.00
PIH/chronic HTN	3 (8.1)	4 (12.5)	.696
Chorioamnionitis	3 (8.1)	6 (18.8)	.285
pPROM	18 (48.6)	15 (46.9)	.883
Antenatal steroid (complete)	27 (73.0)	18 (56.3)	.146
Cesarean section	23 (62.2)	24 (75.0)	.254
Multiple gestations	7 (18.9)	4 (12.5)	.527
Gender, male	21 (56.8)	16 (50.0)	.575
Small for gestational age	4 (10.8)	2 (6.3)	.679
Gestational age, wks	27.7 ± 1.5	27.6 ± 1.7	.853
Birth weight, g	1016.0 ± 172.2	996.9 ± 171.4	.646
5-min Apgar score ≤ 3	1 (2.7)	1 (3.1)	1.00
Use of surfactant	34 (91.9)	30 (93.8)	.767

### 3.2. PDA evaluation

Table 2 shows the PDA evaluations conducted during the study period. In the PT group, PDA was closed in 81.1% (n = 30) of infants at 24 hours after birth, 91.9% at 3 days after birth, and 94.6% at 7 days after birth. The closing rates at 24 hours, 48 hours, 72 hours, and 7 days after birth were significantly higher (*P* < .001) in the PT group than in the non-PT group. Two (5.4%) infants in the PT group and 9 (28.1%) in the non-PT group had moderate-to-large open ductus and were treated with ibuprofen after 7 days of life. Although none of the patients in the PT group experienced reopening of the PDA after confirmation of PDA closure, 2 (6.3%) in the non-PT group did; however this difference was not statistically significant. Four infants who still had open ductus after ibuprofen treatment (one in the PT group, 3 in the non-PT group) underwent surgical ligation.

**Table 2:** Patent ductus arteriosus evaluation.

Characteristics	PT group (n = 37)	non-PT group (n = 32)	*P*
Cumulative no. of closed or tiny PDA at PND 1	30 (81.1)	3 (9.4)	<.001
Cumulative no. of closed or tiny PDA at PND 2	34 (91.9)	4 (12.5)	<.001
Cumulative no. of closed or tiny PDA at PND 3	34 (91.9)	7 (21.9)	<.001
Cumulative no. of closed or tiny PDA at PND 7	35 (94.6)	11 (34.4)	<.001
Moderate-to-large PDA after PND 7	2 (5.4)	9 (28.1)	<.001
Closed or tiny but reopened later (moderate-to-large)	0	2 (6.3)	.211
PDA ligation	1 (2.7)	3 (14.3)	.330

### 3.3. Renal and GI function during the first 3 days of life and enteral feeding progression

Considering the half-life and elimination time of ibuprofen, 72 hours was believed to be sufficient for observation, and the eventual side effects were monitored for 3 days.^[25]^ When prophylactic ibuprofen was administered on the 1st day of life, except for urine output in the 1st 24 hours, no differences were found between the 2 groups in terms of renal function (urine output and serum creatinine concentration) and abdominal distension (Table 3). There was more GI bleeding in the PT group, but the difference was not statistically significant between the 2 groups. The duration of time for feeding to reach 50 mL/kg was significantly shorter in the PT group compared to that in the non-PT group (11.4 ± 4.2 vs 27.7 ± 16.9, *P* < .001).

**Table 3:** Renal and gastrointestinal function during the first 3 days of life and progression of enteral feeding.

Characteristics	PT group (n = 37)	non-PT group (n = 32)	*P*
Urine output (mL/kg/h)			
Day 1	2.4 ± 1.0	3.3 ± 1.2	.005
Day 2	4.2 ± 1.0	4.8 ± 1.2	.055
Day 3	3.6 ± 0.8	4.0 ± 1.1	.230
Serum creatinine (mg/dl)			
Day 1	0.83 ± 0.19	0.82 ± 0.17	.897
Day 2	0.93 ± 0.17	0.89 ± 0.20	.478
Day 3	0.95 ± 0.22	0.97 ± 0.30	.791
Abdominal distension	5 (13.5)	8 (25.0)	.525
Upper gastrointestinal bleeding	7 (18.9)	4 (12.5)	.468
Persistent pulmonary hypertension	0	0	
Age achieved feeding of 50 mL/kg, d	11.4 ± 4.2	27.7 ± 16.9	<.001

### 3.4. Clinical characteristics of the PT and non-PT groups during hospitalization

The early-onset morbidity and clinical characteristics of the 2 groups are compared in Table 4. There were no significant differences in the use of surfactants, administration of initial antibiotics, and early stage morbidity after birth such as pneumothorax and massive pulmonary hemorrhage. Compared with the non-PT group, preterm infants in the PT group were more likely to have a shorter duration of invasive ventilatory support and central venous catheter use, earlier postnatal age of achieving full feeding (100 mL/kg/day), and shorter hospitalization periods. There was no difference in mortality between the 2 groups. In the univariate analysis, the risk of major morbidities such as LOS, NEC, retinopathy of prematurity, and BPD did not differ between the 2 groups, but IVH (≥grade 2) was significantly lower in the PT group. In the multivariate analysis, the odds ratios for IVH (≥grade 2) remained significant after adjustment for confounding variables such as chorioamnionitis, multiple gestations, and the 5-mine Apgar score (Table 5).

**Table 4:** Clinical variables in the PT and non-PT groups.

Variables	PT group (n = 37)	non-PT group (n = 32)	*P*
Use of surfactant	34 (91.9)	30 (93.8)	.767
Initial antibiotics	9 (24.3)	8 (26.7)	.827
Pneumothorax	1 (2.7)	1 (3.1)	1.00
Massive pulmonary hemorrhage	2 (5.4)	4 (12.5)	.405
Duration of invasive ventilatory support, d	4.2 ± 10.0	14.4 ± 16.2	.003
Duration of central venous catheter use, d	21.5 ± 16.2	38.5 ± 16.6	<.001
Age achieved feeding of 100 mL/kg, d	16.1 ± 7.4	34.8 ± 15.1	<.001
Hospital stay, d	69.6 ± 16.1	80.0 ± 23.5	.048
Death	0	2 (6.3)	.211

**Table 5:** Unadjusted and adjusted odds ratios for mortality and major neonatal morbidities.

Outcome	Odds ratio (95% CI)			
	Univariable	*P*	Adjusted^a^	*P*
LOS	0.42 (0.04–4.82)	.483	0.51 (0.04–6.29)	.602
NEC ≥ stage II	–		–	
BPD	0.76 (0.28–2.04)	.586	0.74 (0.26–2.08)	.566
BPD ≥ moderate	0.29 (0.07–1.24)	.095	0.25 (0.06–1.17)	.078
ROP ≥ stage 2	1.43 (0.47–4.35)	.530	1.57 (0.50–4.94)	.444
IVH ≥ grade 2	0.06 (0.01–0.51)	.01	0.05 (0.01–0.45)	.007
IVH ≥ grade 3	0.10 (0.01–0.86)	.036	0.10 (0.01–0.83)	.034

### 3.5. The change of growth parameters from birth to discharge

The growth parameters of the preterm infants who survived and were discharged home in the 2 groups were compared (Table 6). Despite the significantly longer duration of hospital stay in the non-PT group (80.0 ± 23.5 days) than in the PT group (69.6 ± 16.1 days), there was no statistically significant difference in growth parameters (weight, head circumference, height) between the groups. The single-dose prophylactic ibuprofen group demonstrated a higher growth rate in terms of weight, head circumference, and height, and a lower PMA at discharge than the non-PT group.

**Table 6:** Comparison of the growth parameters in the study cohort.

Parameter^a^	PT group (n = 37)	non-PT group (n = 30)	*P*
Birth weight, g	1016.0 ± 172.2	996.9 ± 171.4	.646
HC at birth, cm	25.3 ± 1.9	25.6 ± 1.9	.614
Height at birth, cm	36.2 ± 2.8	36.3 ± 2.6	.882
Weight at discharge, g	2604.8 ± 319.1	2715.3 ± 336.2	.181
HC at discharge, cm	32.5 ± 0.8	33.0 ± 1.2	.07
Height at discharge, cm	46.3 ± 1.8	46.2 ± 1.9	.936
Average daily weight change (weight from birth to discharge/hospital stay), g	24.6 ± 3.6	20.4 ± 3.3	<.001
Average daily HC change (HC from birth to discharge/hospital stay), cm	0.11 ± 0.02	0.09 ± 0.17	<.001
Average daily height change (height from birth to discharge/hospital stay), cm	0.14 ± 0.04	0.12 ± 0.03	.023
PMA at discharge, wks	37.7 ± 1.2	38.8 ± 1.5	.001

## 4. Discussion

PDA occurs in only 57 per 100,000 normal births for full-term infants, but in low BW infants, PDA treatment is required in 55% to 70%, with only 34% of PDA cases closing by itself.^[7,8,26]^ Prophylactic administration of COX inhibitors for 3 days immediately after birth probably decreases the incidence rates of PDA, the need for rescue treatment with COX inhibitors, PDA ligation, and grade 3 or 4 IVH, but is not recommended for prevention due to the increased risks of oliguria, increase in serum creatinine levels, and GI bleeding. All previous studies used 3 doses of COX inhibitors for 3 days, and there were inevitably many side effects.^[11,27]^ In contrast to previous studies, we used only a single dose of a COX inhibitor (ibuprofen) prophylactically within 6 hours of birth. The results of our study demonstrate the efficacy of ibuprofen as prophylaxis for PDAs, showing that administration of a single dose on the 1st day of life effectively reduces the incidence rates of PDA, as compared to previous studies in which ibuprofen was administered for 3 consecutive days. In this study, 81.1% (30/37) of infants in the PT group had a closed or tiny ductus, compared to 9.4% (3/32) in the non-PT group after 24 hours of life, 91.9% compared to 12.5% after 48 hours, 91.9% compared to 21.9% after 72 hours, and 94.6% compared to 34.4% after 7 days of life. This significant reduction in the number of infants with PDA in the PT group corresponds to a significant reduction in the need for rescue therapy for moderate-to-large PDA after 7 days of life. Previous studies of preterm infants born before 28 weeks of gestation showed that the PDA closure rate was approximately 26% at 24 hours, 72–83.1% at 72 hours, and 77% at 7 days after birth when IV ibuprofen was used prophylactically for 3 days after birth. These rates were lower than those in our study, which may be due to the lower GA of the subjects in previous studies.^[14,16,28]^ In the present study, although prophylactic ibuprofen was administered, there were no adverse effects other than a slight decrease in the urine volume on the 1st day. Unlike other studies in which ibuprofen was administered,^[29]^ GI bleeding did not increase, GI function was not lowered, and the time to achieve a feeding of 50 mL/kg/day was significantly faster in the PT group than in the non-PT group. There have been some reports that ibuprofen administration is associated with persistent pulmonary hypertension of the newborn (PPHN),^[30,31]^ but there were no cases of PPHN in this study.

Although a single dose of prophylactic ibuprofen was not associated with neonatal complications including LOS, NEC (≥stage 2), and BPD, it significantly lowered IVH (≥grade 2) and severe IVH (≥grade 3) in this study. A systematic review of the literature found that there was no evidence that prophylactic use of 3 doses of ibuprofen had any benefit in reducing mortality, BPD, or NEC, but reported a reduced risk of severe IVH.^[11]^ There were also studies that 3 doses of prophylactic indomethacin reduced grades 2–4 IVH.^[32–34]^ In a study on the efficacy of ibuprofen in the treatment of PDA, ibuprofen showed results as effective as indomethacin, but with fewer adverse events, because it has less effect on cerebral, kidney, and mesenteric blood flow than indomethacin.^[35,36]^ However, side effects of COX inhibitors are inevitable, and administration of 3 doses of ibuprofen can increase the risk of IVH. In such cases, it may be difficult to distinguish whether the occurrence of IVH is due to the PDA or ibuprofen administration.

Compared to those in the non-PT group, preterm infants in the PT group were more likely to have a lower postnatal age to achieve a feeding of 100 mL/kg/d and a shorter duration of invasive ventilatory support, which allowed them to have a shorter duration of central venous catheter use and reduced lung injury. At our center, feeding begins with the mother’s milk or donor human milk. If preterm infants at 2 weeks of life can tolerate volumes >100 mL/kg/day, human milk fortifier is added to human milk. Therefore, the growth (weight, head circumference, and length) of preterm infants who reach full feeding sooner will inevitably be faster. Preterm infants have more nutritional needs during the neonatal period when their nutritional requirements are inherently high to match the high rate of nutrient accumulation in utero.^[37]^ Poor weight gain, although not a disease, may result in numerous medical conditions in preterm infants, particularly neurodevelopmental delay.^[38]^ Although there was no significant difference in the incidence of LOS and BPD between the 2 groups in our study, central venous catheter and invasive ventilation are causal factors for sepsis and BPD; therefore, removing them as soon as possible will help reduce morbidities in preterm infants.^[39,40]^

The study was limited by data from a single institution, small sample size, and the lack of long-term neurodevelopmental outcomes after NICU discharge. As this was a retrospective study, there were limitations to the analysis. To the best of our knowledge, there are no clinical reports of single-dose prophylactic IV ibuprofen administration, and a prospective study and clinical evaluation with more study groups are needed. Studies on the timing and method of administration of ibuprofen, its clinical course and its long-term effects should be conducted.

## 5. Conclusions

In moderately mature preterm infants with high rates of spontaneous PDA closure and a low incidence of IVH, prophylactic COX inhibitor therapy using 3 doses may not be necessary and may produce substantial side effects. Single-dose prophylactic ibuprofen may be a suitable treatment for very premature infants born before 30 weeks of gestation, given that it has a similar PDA closure rate and reduced IVH incidence as 3-dose prophylactic ibuprofen therapy, while avoiding the risks of well-known side effects associated with the drug.

## Author contribution

Conceptualization: Sung-Hoon Chung. Data curation: Chae Young Kim. Formal analysis: Sung-Hoon Chung. Investigation: Chae Young Kim, Sung-Hoon Chung. Methodology: Sung-Hoon Chung. Supervision: Sung-Hoon Chung. Validation: Chae Young Kim. Writing—original draft: Chae Young Kim. Writing—review, and editing: Sung-Hoon Chung.

## Footnotes

a For chorioamnionitis, multiple gestation, and 5-min Apgar score ≤ 3 in the study.

a Data from the cases died were excluded.
